# Effect of ultrasound-guided stellate ganglion block on inflammatory cytokines and postoperative recovery after partial hepatectomy: a randomised clinical trial

**DOI:** 10.1186/s12871-023-02392-7

**Published:** 2024-01-02

**Authors:** Wei-long Lao, Shuang Sang, Li-cai Huang, Sheng-hua Yi, Mo-chi Guo, Hui-min Dong, Guo-zhong Zhou, Zhong-hua Chen

**Affiliations:** 1 Department of Anesthesia, Shaoxing People’s Hospital, Shaoxing, China; 2 Shaoxing University School of Medicine, Shaoxing, China; 3 Clinical laboratory, Shaoxing People’s Hospital, Shaoxing, China

**Keywords:** Enhanced recovery after surgery (ERAS), Inflammatory cytokines, Partial hepatectomy, Stellate ganglion, Ultrasound guidance

## Abstract

**Background:**

Stellate ganglion block (SGB) has been shown to reduce perioperative complications in various surgeries. Because laparoscopic techniques and instruments have advanced during the past two decades, laparoscopic liver resection is being increasingly adopted worldwide. Lesser blood loss, fewer postoperative complications, and shorter postoperative hospital stays are the advantages of laparoscopic liver resection, as compared to conventional open surgery. There is an urgent need for an effective intervention to reduce perioperative complications and accelerate postoperative recovery. This study investigated the effect of ultrasound-guided SGB on enhanced recovery after laparoscopic partial hepatectomy.

**Methods:**

We compared patients who received SGB with 0.5% ropivacaine (group S) with those who received SGB with 0.9% saline (group N). A total of 58 patients with partial hepatectomy were enrolled (30 S) and (28 N). Before induction of anesthesia, SGB was performed with 0.5% ropivacaine in group S and 0.9% saline in group N. Main outcome: Comparison of serum inflammatory cytokines concentration at each time point.

**Results:**

Main outcome: When comparing IL-6 and IL-10 concentrations among groups, group S showed less variation over time compared to group N. For comparison between groups, the serum IL-6 concentration in group S was lower than that in group N at 6 and 24 h after operation (*P* < 0.01), and there was a significant linear relationship between serum IL-6 concentration at 24 h after operation and hospitalization situation.

**Conclusions:**

Ultrasound-guided SGB can stabilize perioperative inflammatory cytokines plays a positive role in the enhanced recovery of patients after laparoscopic partial hepatectomy. The serum IL-6 level within 24 h after surgery may be used as a predictor of hospitalization.

**Trial registration:**

The study was registered at the ClinicalTrials.gov (Registration date: 13/09/2021; Trial ID: NCT05042583).

## Introduction

Laparoscopic partial hepatectomy is an increasingly essential surgical therapy for liver, bile duct, and other illnesses, since the benefits of minimally invasive hepatectomy are more validated and its indications are steadily expanded [1–4]. Hepatectomy carries many risks, including a longer operation time, more blood loss during operation, a large amount of fluid transfer, a high risk of hypotension, hepatic portal occlusion, ischemia-reperfusion injury, and more postoperative complications than other abdominal operations, even when performed using minimally invasive techniques. Moreover, patients may have significant liver function impairment due to the effects of anticancer medications, making it even more important to perform careful perioperative treatment.

To create its therapeutic effects, stellate ganglion blockade is known to have a function in controlling the autonomic nerve system, endocrine system, and immunological system, as is common knowledge. All three of these systems—the autonomic nervous system, the endocrine system, and the immune system—play a significant role in a speedier recovery for patients following surgery [5–7]. There are currently only a few numbers of research on the use of stellate ganglion block to improve postoperative recovery.

In this investigation, stellate ganglion block was used because of its ability to control not only the autonomic nervous system (regulating gastrointestinal function [8], sleep disorder [9], hemodynamic stability [10], prevention and treatment of arrhythmias [11]), but also the endocrine and immune systems (regulating the production of acute excessive inflammatory reactions caused by various reasons, such as “cytokine storms” caused by inflammatory cascade reactions [5, 12]). With the modulation of inflammatory cytokines as the primary outcome, we tested it on patients having elective laparoscopic partial hepatectomy and analyzed its effect on postoperative tissue harm and improved postoperative recovery.

## Materials and methods

### Ethics

The Ethics Committee of Shaoxing People’s Hospital in Shaoxing, China (Chairperson Prof Bao-chun Lu) approved this study (Ethical Committee N°2020-K-Y-185-01) on 24 July 2020. The study was successfully registered in ClinialTrials.gov on 13 September 2021 (ID: NCT05042583), and is continuing in progress, with the informed agreement of patients and their families. The first patient enrolment date was January 1, 2021, and 58 patients who had undergone elective laparoscopic partial hepatectomy at Shaoxing People’s Hospital had been included in the study. The following criteria must be met: (1) patients who underwent elective laparoscopic hepatectomy; (2) Classification of NYHA cardiac function: I or II; (3) ASA-PS ≤ III; (4) Age between 18 and 75 years old. Conditions for exclusion: (1) Non-laparoscopic hepatectomy patients; (2) Patients younger than 18 or older than 75 years old; (3) Classification of NYHA heart function ≥ III; (4) ASA-PS > III; (5) Allergy to local anesthetics; (6) Patients with immune diseases or immunosuppression; (7) Patients with severe mental illness who are unable to cooperate with SGB; (8) Patients with abnormal neck anatomy; (9) Patients refused to participate in the researcher; (10) The coagulation function is obviously abnormal; (11) There are insurmountable difficulties in follow-up and specimen collection.

### Study design

This study is a single-center, prospective, randomized controlled, and blinded investigation. Patients were divided into the experimental group (group S) and the control group (group N) on the morning of the surgery. The group assignment was decided by a computer-generated random sequence table, and the random sequences were held in sealed opaque envelopes, which were opened by the observer before the anesthesia. The group assignment and anesthesia were managed by investigators who were not involved in postoperative follow-up, and the surgeon, patient, and investigator responsible for the operation and postoperative follow-up were unaware of the anesthetic approach. In group S, 0.5% ropivacaine 6mL was utilized for stellate ganglion block under ultrasound guidance before general anesthesia. Patients in group N got stellate ganglion block under ultrasound guidance with 0.9% saline 6mL before general anesthesia.

### Stellate ganglion block procedure and its successful performance

A high-frequency linear ultrasound probe (12 MHz) is applied while the patient is supine with his head tilted to the opposite side. The probe is positioned at the level of the notch in the cricoid cartilage on the patient’s left side, and its direction is 30–45 degrees off the sagittal plane of the neck. Besides the carotid artery, jugular vein, anterior scalene muscle, and long neck muscle, several nearby structures, like the C6 cone and its distinctive “double-peak” transverse process, can be seen and identified as well. And do local routine disinfection. The needle is put outside the carotid artery and into the loose connective tissue on the longus cervical muscle, avoiding essential blood arteries, nerves, and organs. There is no resistance to pressing the drug or pumping blood and gas back. Diffusion of 6 ml of medicinal liquid blocks stellate ganglions. The common indicators of a successful block are Horner’s syndrome on the operative side, negative sweat test, and noticeable skin temperature increase in the dominating area.

### Anesthesia program

The patient next punctured the left radial artery to begin invasive blood pressure monitoring after the block. Nearly all patients in our previous study developed Horner’s syndrome within 10 min after stellate ganglion block, similar to the results reported in previous related studies [13], so we decided to induce anesthesia after 10 min of block with midazolam 0.02 mg/kg, etomidate 0.1–0.3 mg/kg, cis-atracurium 0.3 mg/kg and sufentanil 0.3–0.5 µg/kg, and to use Drager anesthesia machine for mechanical ventilation after tracheal intubation. Intravenous infusion of remifentanil 0.1–0.2 µg/kg/min, cis-atracurium 1 µg/kg/min, propofol and sevoflurane were utilized to maintain the anesthetic depth (BIS 40–60). The administration of cis-atracurium was halted 30 min before the completion of the operation, and all patients were given ondansetron 8 mg to reduce vomiting following the operation. The anesthesiologist judged that all maintenance drugs should be terminated 5 min before the completion of the operation. If the judgment was mistaken, propofol should be given adequately to continue the operation. After the operation, all patients were given patient-controlled intravenous analgesia (PCIA, 100 µg Sufentanil Citrate and 150 mg Flurbiprofen Axetil) and then resuscitated in the post anesthesia care unit (PACU). The indications for transferring out of PACU conform to the guidelines of Miller Anesthesiology, 7th edition.

### Outcomes

#### Main outcome

Inflammatory cytokines: 8 ml blood samples were collected from patients before SGB (T0), 1 h (T1), 3 h (T2), 6 h (T3), 24 h (T4) and 72 h (T5) after operation. All blood samples were centrifuged at 3000 rpm for 10 min, and the separated serum was stored in a refrigerator at -80 °C and thawed until the need for detection. Serum inflammatory cytokines IL-2, IL-6, IL-10, TNF-α and IFN-γ were detected by cytometric bead array (CBA).

#### Secondary outcome

Related events during and after the operation: record the operation time, blood loss during the operation, blood transfusion cases during the operation, vasoactive drugs used during and after the operation (, extubation time after the end of anesthesia, postoperative complications and Clavien-Dindo Classification, additional tramadol analgesia cases in the ward within 48 h, the first postoperative exhaust time, postoperative fluid intake time, semi-fluid time, the number of liver segments involved in hepatectomy, and postoperative pathology were recorded through the hospital medical record system and postoperative follow-up. Hemodynamics: The hemodynamic changes at baseline before operation, immediately before block, 15 min after block, 35 min after block, and out of PACU were recorded. Transaminase other inflammatory markers: The variations of alanine transaminase (ALT), aspartate transaminase (AST), C-reactive protein (CRP) and white blood cell (WBC) before operation, post operation day 1 (POD1), post operation day 3 (POD3) and post operation day 6 (POD6) were recorded. Post-operation hospitalization: The hospitalization costs and length of stay from the day of operation were recorded.

### Statistical analysis

The sample size was determined using the PASS15 program, and information for the independent variable, inflammatory cytokines, was gathered from the published literature [14]. Sample sizes of 24 in group S and 24 in group N were determined using the two independent sample mean test standard, the bilateral = 0.05, and a confidence degree of 90%. Due to the possibility of a sample loss of more than 20% during the experiment, a total of 33 patients meeting the criteria for inclusion in each group were sought out.

The measurement data involved in this study, a single sample K-S normal distribution test. The measurement data conforming to the normal distribution is expressed by the Mean ± SD ($\bar {\rm x}$ ± s); T-test was used to examine the differences between groups, and repeated measurement ANOVA was used to compare the differences at different time points within groups. Data from non-normal distributions are expressed using the median (interquartile range), and the Wilcoxon rank sum test is used to examine differences across groups; for repeated-measures data, the generalized estimation equation is employed. The percentages from the categorical data are put through a test using either the chi-square or Fisher’s exact statistic. SPSS 25.0 was used for all statistical analysis. *P* < 0.05 is statistically significant. Carry out an R-value study of correlation using a standard linear regression model. Statistics are mapped using GraphPad Prism.

## Results

### Study participants

**Fig. 1:**
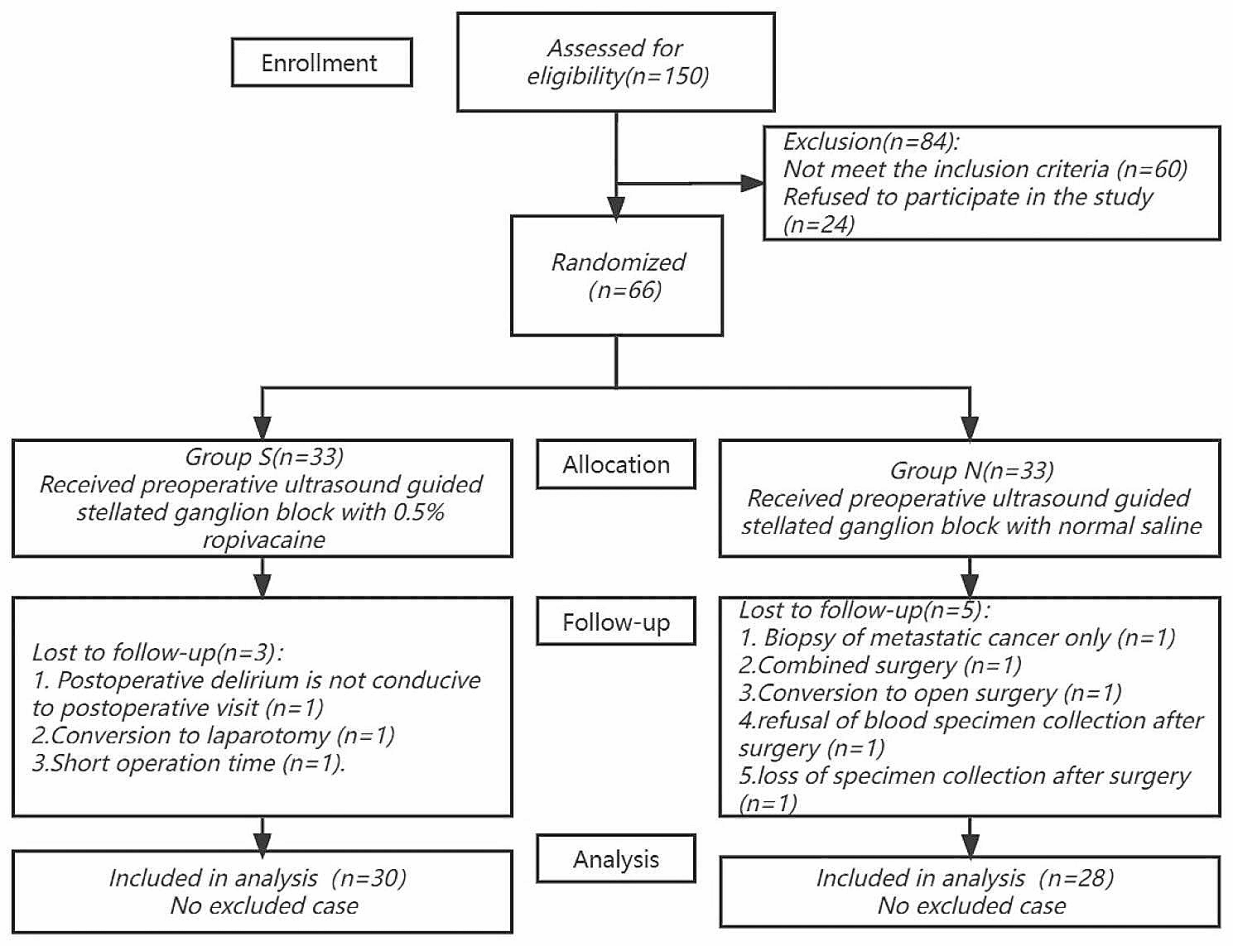
CONSORT flow diagram of participants in the study

Between January 2021 and June 2022, we evaluated 150 patients who were eligible for laparoscopic partial hepatectomy. In the end, 66 people were randomly allocated after 60 were excluded and 24 declined to take part in the study. There were 66 patients total, evenly split between two groups of 33. In the follow-up, Three patients were eliminated from group S in the follow-up: one had postoperative delirium, making a postoperative visit unfeasible; one was converted to laparotomy during the surgery; and one had a short operation length. Five patients were eliminated from group N: one who only had a biopsy of metastatic cancer during the procedure, one who needed a combination operation, one who converted to laparotomy during the operation, one who declined to collect blood samples after the operation, and one who failed to collect blood samples after the operation. Therefore, 58 patients were assessed for inclusion in the final analysis (Fig. 1). The baseline parameters, operation time, number of liver segments involved in the operation, and postoperative pathology were similar between the two groups, and were comparable between the two groups (Tables 1 and 2).

**Table 1:** Intraoperative and postoperative procedure-related events

Event	Group S(*n* = 30)	Group N(*n* = 28)	Statistical value	*P* value
Time of operation (min)	180.00(130.00 to 251.25)	235.00(171.25 to 320.00)	Z=-1.736	0.083
Intraoperative blood loss(ml)	300.00(142.50 to 450.00)	300.00(100.00 to 450.00)	Z=-0.165	0.8869
Intraoperative blood transfusion	9(30.0%)	15(53.6%)	χ2 = 3.317	0.069
Plasma only	4(13.3%)	7(25%)		
Erythrocyte only	0(0%)	0(0%)		
Plasma + Erythrocytes	5(16.7%)	8(28.6%)		
Vasoactive drug intervention was required during the operation	14(43.3%)	20(71.4%)	χ2 = 4.661	0.031
Single dosage	10(33.3%)	15(53.6%)		
Pump injection	3(10%)	5(17.9%)		
Postoperative vasoactive drug intervention was required	0	0		
Extubation time after the end of anesthesia (min)	20.00(10.00 to 32.50)	15.00(7.00 to 30.00)	Z=-1.528	0.127
Postoperative complications Clavien-Dindo Classification			χ2 = 1.809	0.179
I	3(10%)	2(7.1%)		
II	14(46.7%)	15(53.6%)		
IIIa	3(10%)	6(21.4%)		
IIIb	0	0		
IVa	0	0		
IVb	0	0		
V	0	0		
Additional cases of tramadol analgesia were required within 48 h	6(19.4%)	2(7.1%)	χ2 = 1.077	0.299
Use once	4(13.3%)	2(7.1%)		
Use twice	2(7.1%)	0(0%)		
The first postoperative exhaust time (h)	44.25(29.00 to 60.00)	50.00(40.00 to 68.00)	Z=-1.277	0.202
Postoperative fluid intake time (h)	23.50(18.00 to 45.00)	41.00(22.00 to 44.00)	Z=-1.712	0.087
Postoperative semisolid diet intake time (h)	64.50(42.38 to 69.50)	67.00(48.00 to 75.00)	Z=-0.981	0.326
Number of hepatic segments involved in liver resection			χ2 = 4.481	0.236
1 hepatic segment	15(50%)	10(35.7%)		
2 hepatic segments	10(33.3%)	7(25%)		
3 hepatic segments	3(10%)	9(32.1%)		
4 hepatic segments	2(6.7%)	2(7.1%)		
Postoperative pathology				
Benign /Malignant	18/12	13/15	χ2 = 1.072	0.300
Hepatocellular carcinoma	9(30%)	10(35.7%)		
Intrahepatic Cholangiocarcinoma	2(6.7%)	2(7.1%)		
Benign liver tumors	7(23.3%)	1(3.6%)		
Intra-and extrahepatic bile duct stone	4(13.3%)	10(35.7%)		
Atrophy of liver cirrhosis	0(0%)	1(3.6%)		
Liver metastases	1(3.3%)	3(10.7%)		
Hepatic hemangioma	7(23.3%)	1(3.6%)		
Pringle maneuvers were used	23(76.7%)	22(78.6%)	χ2 = 0.030	0.862

Values are median (range) or number (proportion)

**Table 2:** Baseline characteristics of the trial patients

Characteristic	Group S(*n* = 30)	Group N(*n* = 28)	Statistical value	*P* value
Gender (male/female)	19/11	18/10	χ2 = 0.006	0.940
Age (years old)	60.23 ± 10.56	63.43 ± 10.00	t=-1.181	0.242
BMI	24.36 ± 2.60	23.37 ± 3.08	t = 1.320	0.192
ASA score			χ2 = 0.291	0.864
1	2(6.7%)	1(3.6%)		
2	25(83.3%)	24(85.7%)		
3	3(10.0%)	3(10.7%)		
4	0	0		
Previous hepatectomy	1(3.3%)	1(3.6%)	χ2<0.001	>0.999
Postoperative interventional therapy was needed	8(26.7%)	9(32.1%)	χ2 = 0.210	0.647
Postoperative Chemotherapy is Required	7(23.3%)	4(14.3%)	χ2 = 0.771	0.380

Values are mean ± SD or number (proportion)

### Main outcome

**Fig. 2:**
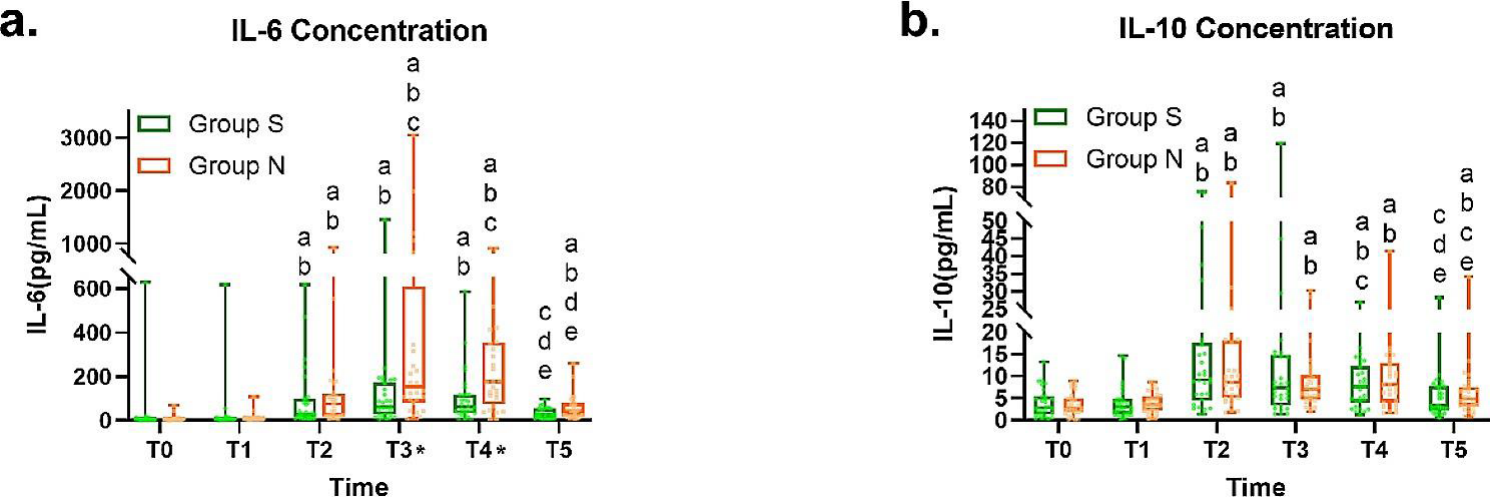
**(a)** Box line scatter diagram of the concentration change of IL-6 in the group S and group N at each time point; **(b)** Box line scatter diagram of the concentration change of IL-10 in group S and group N at each time point (The box is the quartile separation, the ends of the extension line are the maximum and minimum values, and the scatter points are the distribution of the data) a: There is statistical difference with before block; b: There was a statistical difference with 1 h after the operation; c: There was a statistical difference with 3 h after the operation; d: There was a statistical difference with 6 h after the operation; e: There was a statistical difference with 24 h after the operation started; *: There is a statistical difference between group S and group N (*P* < 0.05)

We detected the levels of five inflammatory cytokines such as IL-2, IL-6, IL-10, TNF-α and IFN-γ in patients before SGB (T0), 1 h (T1), 3 h (T2), 6 h (T3), 24 h (T4) and 72 h (T5) after the start of operation. Figure 2a shows that group S and group N had different IL-6 concentration distributions at each time point (*P* < 0.001). At T3 and T4, group S [62.82 (26.81 to 173.19), 62.37 (33.50, 117.62)] had lower IL-6 levels than group N [153.00 (82.88 to 610.11), 175.95 (74.05 to 354.83), *P* < 0.01]. At T5, there was no significant difference between T0 [4.67 (2.9 to 9.66)] and T5 [26.57 (11.36 to 55.21)] in group S (*P* = 0.652), while there was still significant difference between T0[5.54 (3.06 to 7.53)] and T5 [43.99 (20.16 to 77.87)] in group N (*P*<0.001). As shown in Fig. 2b, the intra-group comparison of IL-10 concentrations in group S and group N showed substantial differences in inflammatory factor concentrations at each time point (*P* < 0.001), although there was no statistical difference between groups (*P* > 0.05). At T5, there was no significant difference between T0 [2.89 (1.36 to 5.46)] and T5[3.36 (2.24 to 7.73)] in group S (*P* = 0.115), while there was still significant difference between T0 [2.82 (1.87 to 4.98)] and T5 [4.97 (3.18 to 7.63)] in group N (*P* = 0.002).

### Secondary outcome

#### Intraoperative situation

**Fig. 3:**
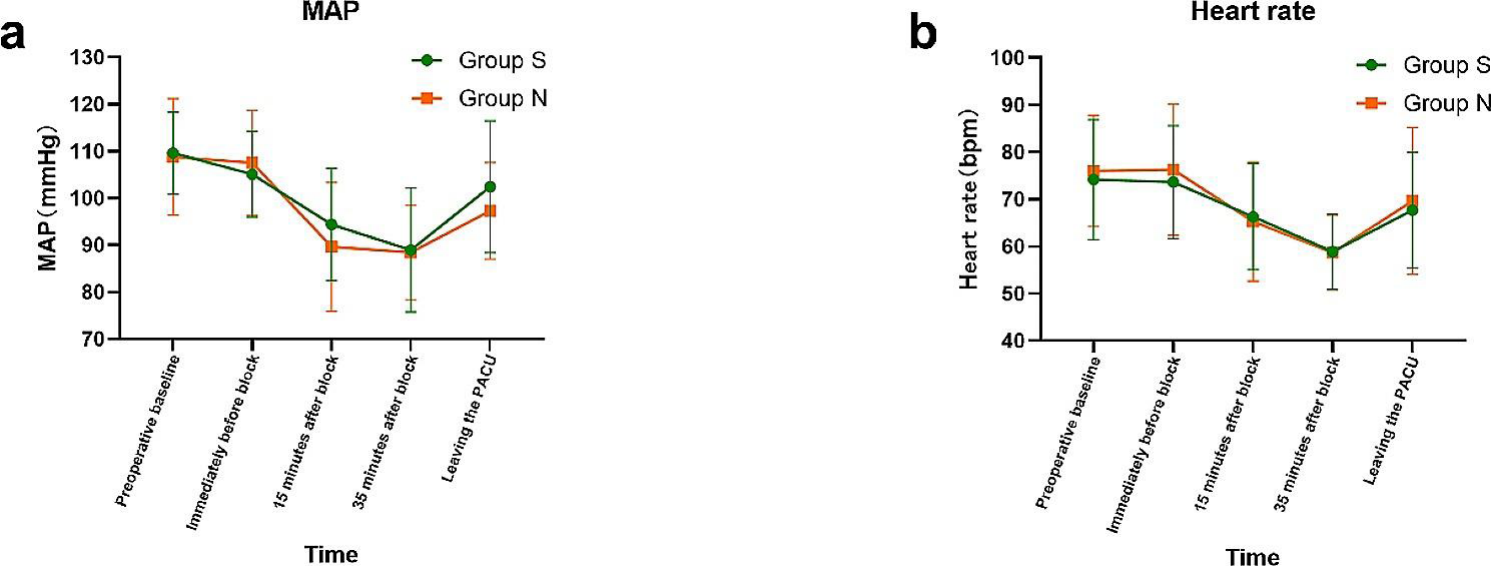
**(a)** Trend plot of mean arterial pressure (MAP) in group S and group N at each time points; **(b)** Trend plot of heart rate in group S and group N at each time points (The center point is the mean, and the two ends of the extension line are standard deviations)

The two groups had similar bleeding and blood transfusions, but vasoactive medication use was statistically different. Group S [14 (43.3%)] had fewer vasoactive drug instances than group N [20 (71.4%)] (*P* = 0.031) (Table 1). At the same time, we recorded some hemodynamic data (Fig. 3). Although there were significant differences in intra-group comparison at each time point (*P* < 0.001), the variance of group S (*F* = 19.443) was smaller than that of group N (*F* = 24.318) in terms of the fluctuation of MAP, and the dispersion of data was smaller and the fluctuation of blood pressure was smaller.

**Fig. 4:**
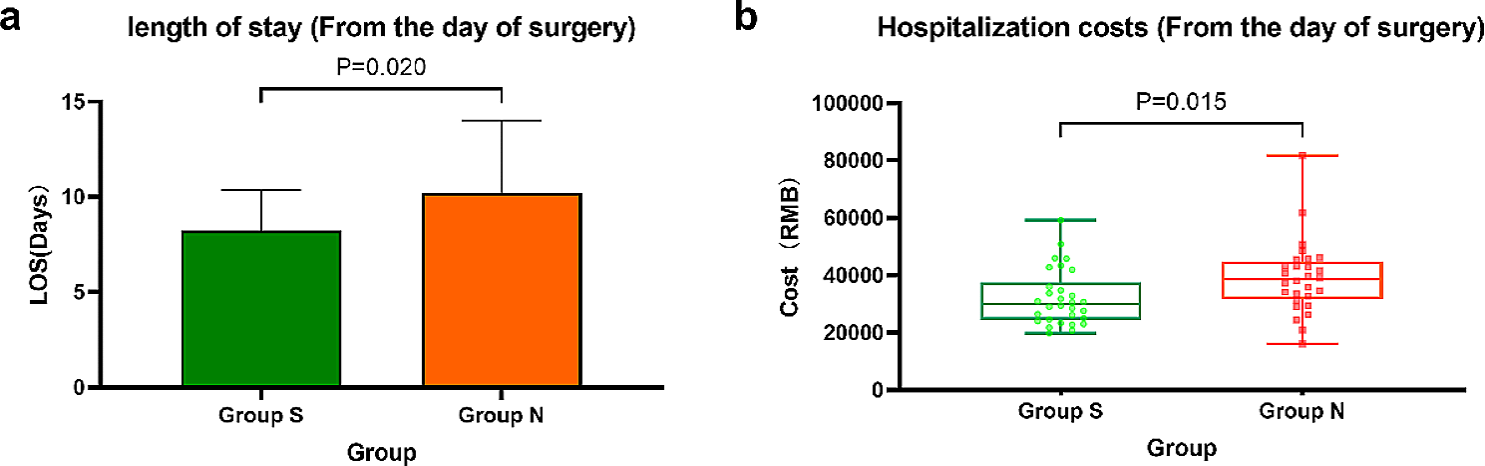
**(a)** Histogram of comparison of length of stay (calculated from the day of operation) between Group S and Group N (expressed as average and standard deviation); **(b)** Box line scatter diagram of comparison of hospitalization costs (calculated from the day of operation) between Group S and Group N (The box is the quartile separation, the ends of the extension line are the maximum and minimum values, and the scatter points are the distribution of the data)

#### Complications 30 days after the operation

Both groups had similar Clavien-Dindo Classification of Postoperative Complications scores (Table 1). Only the number of instances with greater drainage fluid in the abdominal cavity in Group S was lower than in Group N (*P* = 0.047), while the rest of the problems were identical (Table 3). We detected no ultrasound-guided stellate ganglion block complications.

**Table 3:** Complications within 30 days after the operation

Event	Group S(*n* = 30)	Group N(*n* = 28)	χ2 value	*P* value
Incision infection	1(3.3%)	0(0%)	<0.001	>0.99
Fever	7(23.3%)	11(39.3%)	1.722	0.189
Nausea	1(3.3%)	1(3.6%)	<0.001	>0.99
NRS ≥ 4 for pain	5(16.7%)	2(7.1%)	0.503	0.478
Bile leakage (no treatment required)	0(0%)	3(10.7%)	1.557	0.212
Acute Kidney Injury (Transient)	1(3.3%)	2(7.1%)	0.004	0.951
Chronic Pain	1(3.3%)	0(0%)	<0.001	>0.99
Atrial fibrillation	1(3.3%)	0(0%)	<0.001	>0.99
Gastrointestinal dysfunction	13(43.3%)	8(28.6%)	1.366	0.242
More drainage fluid in the abdominal cavity	4(13.3%)	10(35.7%)	3.962	0.047
Culture of drainage fluid suggested abdominal infection	3(10.0%)	6(21.4%)	0.703	0.402
Pulmonary infection	1(3.3%)	2(7.1%)	0.004	0.951
Bile leakage (treatment required)	1(3.3%)	0(0%)	<0.001	>0.99
Pleural effusion (moderate)	3(10.0%)	6(21.4%)	0.703	0.402

Values are number (proportion)

#### Hospitalization situation

**Fig. 5:**
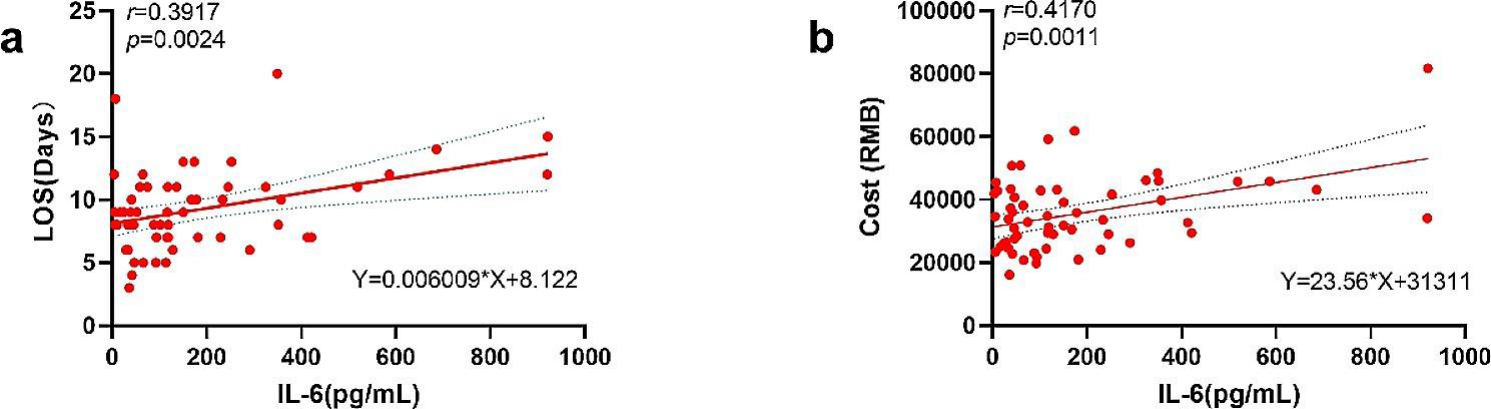
**(a)** Correlation between length of stay (calculated from the day of operation) and IL-6 concentration at T4; **(b)** Correlation between hospitalization expenses (calculated from the day of operation) and IL-6 concentration at T4 (*n* = 58, dotted line is 95% confidence interval)

Group S had a shorter length of stay (calculated from the day of operation) (8.2 ± 2.1, 10.2 ± 3.8, *P* = 0.020) and lower hospitalization costs (calculated from the day of operation) (38622.35 (31676.04 to 44861.15), *P* = 0.015) than group N. (Fig. 4). A simple linear regression analysis was made between IL-6 concentrations at the time points of the primary outcome at which there was a between-group difference and the hospitalization situation (Fig. 5). The length of stay (calculated from the day of operation) was moderately significantly correlated with the concentration of IL-6 at T4 (*r* = 0.3917; *P* = 0.0024; *n* = 58; Fig. 5a), as were the hospitalization expenses (calculated from the day of operation) that were moderately significantly correlated with the concentration of IL-6 at T4 (*r* = 0.4170; *P* = 0.0011; *n* = 58; Fig. 5b). However, the length of stay (calculated from the day of operation) had little correlation with the concentration of IL-6 at T3 (*r* = 0.2761; *P* = 0.0358), and hospitalization expenses (calculated from the day of operation) had no correlation with the concentration of IL-6 at T3 (*r* = 0.1938; *P* = 0.1450).

#### Liver function

There was no significant difference in the effect on liver function between the two groups (Table 4).

**Table 4:** Comparison of transaminases

Event		Preoperative	POD1	POD3	POD6	*P* value
ALT (U/L)	Group S(*n* = 30)	23.6(16.08 to 40.9)	178.2(123.68 to 353.85) a	137.15(79.53 to 210.3) a	64.9(46.53 to 108.3) abc	<0.01
	Group N(*n* = 28)	28.75(14.95 to 39.95)	169.75(110.58 to 302.05) a	127.7(74.68 to 242.5) a	61(38.63 to 95.85) bc	<0.01
	Z value	-0.047	-0.467	-0.319	-0.576	
	*P* value	0.963	0.641	0.75	0.565	
AST(U/L)	Group S(*n* = 30)	23.2(19.8 to 30.85)	165.05(114.28 to 330.2) a	66.15(31.55 to 112.75) ab	25.9(17.8 to 51.33) bc	<0.01
	Group N(*n* = 28)	30.1(20.1 to 40.58)	201.8(121.25 to 353.45) a	62.1(40.45 to 134.38) ab	30(22.13 to 46.48) bc	<0.01
	Z value	-1.611	-0.42	-0.288	-0.607	
	*P* value	0.107	0.674	0.773	0.544	

Values are median (range)

a: There is a statistical difference with before operation, b: There is a statistical difference with POD1, c: There is a statistical difference with POD3.

#### CRP and WBC

The level of CRP in group S on day 6 after surgery was found to be significantly lower compared to that in group N [29.97 (21.94 to 63.33), 46.23(35.27 to 65.22), *P* = 0.038] (Table 5). There was no significant difference in the effect on WBC between the two groups (Table 5).

**Table 5:** Comparison of CRP and WBC

Event		Preoperative	POD1	POD3	POD6	*P* value
CRP (M (P25, P75), mg/L)	Group S(*n* = 30)	1.27(0.50 to 4.10)	32.97(20.86 to 66.90) a	86.80(47.21 to 130.22) ab	29.97(21.94 to 63.33) ac	<0.01
	Group N(*n* = 28)	1.79(0.80 to 15.83)	31.99(20.30 to 59.19) a	105.15(83.52 to 173.55) ab	46.23(35.27 to 65.22) ac	<0.01
	Z value	-1.408	-0.436	-1.572	-2.07	
	*P* value	0.159	0.663	0.116	0.038	
WBC (M (P25, P75),10^9/L)	Group S(*n* = 30)	5.25(4.40 to 6.94)	11.77(9.05 to 14.56) a	9.03(6.18 to 10.97) a	6.64(5.42 to 8.06) bc	<0.01
	Group N(*n* = 28)	5.20(4.49 to 6.30	11.50(9.25 to 14.35) a	9.35(7.90 to 11.93) a	7.08(5.86 to 8.78) bc	<0.01
	Z value	-0.233	-0.062	-0.996	-1.089	
	*P* value	0.815	0.95	0.319	0.276	

Values are median (range)

a: There is a statistical difference with before operation, b: There is a statistical difference with POD1, c: There is a statistical difference with POD3

## Discussion

Ultrasound-guided stellate ganglion block is becoming increasingly popular, and perioperative investigations are increasing [8, 15, 16]. This prospective randomized controlled trial found that ultrasound-guided stellate ganglion block (SGB) plays a role in the management of inflammatory cytokines after elective laparoscopic partial hepatectomy. Group S had much lower levels of the pro-inflammatory cytokine IL-6 at 6 and 24 h after surgery, and it recovered faster. At 72 h, there was no significant difference from before the block in group S, but the level in group N was still significantly higher than before the block, and the level of IL-6 in group S was significantly more stable than that in group N from rise to recovery. Although there was no significant difference in the anti-inflammatory cytokine IL-10 between the two groups at each time point, 72 h after the operation, IL-10 in group S was no different from that before the block, and IL-10 in group N was still much greater. As a result, SGB reduces pro-inflammatory cytokines following laparoscopic partial hepatectomy, minimizing tissue damage.SGB reduces the release of pro-inflammatory cytokines, which may explain the early recovery of anti-inflammatory cytokines. The lack of substantial variations in anti-inflammatory cytokines between groups may be due to the hepatic portal block’s mild ischemia-reperfusion damage and low proinflammatory cytokine production. After all, anti-inflammatory cytokines play a role by inhibiting excessive inflammatory responses, thus limiting the adverse effects induced by excessive inflammation [17]. At the same time, CRP, another significant indicator of inflammation dependent on IL-6 expression, is associated with inflammatory liver damage and liver cancer progression. CRP in group S was significantly lower than that in group N on the sixth day after surgery, and there was no statistical difference when IL-6 reached its peak, which may be related to factors such as the longer delay difference in CRP change and the effect of liver resection on liver release of CRP. In terms of the inflammatory response, Ying Li et al. found that stellate ganglion block could increase CD4 + T cell activity and decreased IL-2, IL-4, and TNF- stimulated TIPE2 production, hence increasing rat survival after severe hemorrhagic shock [6]. In 2020, Eugene Lipov et al. put forward a hypothesis and gave some evidence to show that stellate ganglion block may regulate immune inflammation by regulating sympathetic innervation of primary (thymus, bone marrow) and secondary immune organs (such as the spleen, lymph nodes, and mucosa-associated lymphoid tissues), and it finally inhibits the production of pro-inflammatory cytokines (like IL-1, IL-6, TNF-α, etc.) while increasing the production of anti-inflammatory cytokines (such as IL-4, IL-10, IL-13, etc.) [18]. The findings of these other researches are consistent with ours. Although blocking the stellate ganglion does decrease pro-inflammatory cytokine production and increase anti-inflammatory cytokine production, the mechanism by which this occurs has not been investigated in this work.

Secondly, we chose multiple time points (preoperative baseline, immediately before the block, 15 min after the block, 35 min after the block, and out of the resuscitation room) to monitor the hemodynamics for safety reasons because early stellate ganglion block is a type of cervical sympathetic nerve block and there are not many studies on its application in general anesthesia before. The 15 min after the block was chosen mainly because of the large hemodynamic fluctuations during induction. The 35 min after the block was chosen primarily to observe the hemodynamic effects of stellate ganglion block in the presence of a long waiting period for the start of the operation. According to our findings, the application of SGB in laparoscopic partial hepatectomy will not only have no negative impact on hemodynamics due to blocking sympathetic nerves, but also stabilize the fluctuation of blood pressure during the operation. This may be one of the reasons why vasoactive drugs are used less in group S, where blood pressure is relatively stable, so the number of cases requiring vasoactive drugs is naturally small. This is consistent with the findings of Yong-Quan Chen et al., who discovered that stellate ganglion block stabilizes hemodynamics in elderly patients undergoing carbon dioxide pneumoperitoneum [13].

Furthermore, with regard to the guidance of both Diagnosis Related Groups (DRGs) and The Chinese Expert Consensus on Enhanced Recovery after Hepatectomy (2017 edition), our study statistics from the day of surgery shows that patients in group S exhibited a reduced length of hospital stay and incurred lower hospital expenses compared to those in group N. Although it has been reported that stellate ganglion block can reduce the surgical inflammatory response, reduce surgical stress [8], promote postoperative recovery of gastrointestinal function [19], relieve postoperative pain and reduce arrhythmia [7], the effect on hospital stays has been reported on very seldom. Since IL-6 levels at T3 and T4 varied across groups, we used a simple linear regression analysis to determine whether or not there was a link between the length of stay and hospital costs of the two groups of patients on the day of the surgery and the primary result. According to our findings, stellate ganglion block has a significant impact on the length of stay (calculated from the day of operation) and hospitalization costs (calculated from the day of operation) of patients by influencing the change in IL-6 concentration at T4, and there is a certain linear relationship. This might provide a theoretical study for selecting the concentration of IL-6 24 h after the operation as a visual evaluation index of the prognosis after SGB. After all, according to the existing literature on SGB and inflammatory response, SGB can improve postoperative cognitive dysfunction, and its mechanism may be through the regulation of silent information regular 1 (SIRT1) -mediated neuroinflammation after SGB and correcting white matter damage to improve postoperative cognitive dysfunction [20–22]. SGB can improve postoperative atrial fibrillation. The mechanism may be that it can regulate the balance of autonomic nervous system and reduce systemic and local inflammatory response represented by IL-6, and finally improve postoperative atrial fibrillation [23–25]. SGB improves postoperative gastrointestinal function, and its mechanism may be to regulate the excessive release of systemic and local pro-inflammatory factors to improve postoperative gastrointestinal function [20, 26]. SGB can relieve postoperative inflammatory pain. In addition, inflammatory cytokines have been shown in the literature to influence the occurrence and recovery of postoperative delirium [27], perioperative hemodynamics [28], postoperative liver disease prognosis [28], postoperative wound prognosis and revascularization [29], and other important indicators of surgical recovery. As we all know, TNF-α and IL-6 are the main triggers of inflammatory factor storms within a few minutes after the beginning of hepatectomy, and most liver operations require hepatic portal occlusion [30], which will cause a certain degree of ischemia-reperfusion injury, and ischemia-reperfusion injury will produce a large number of inflammatory cytokines [17, 31]. Another interesting point is that IL-6 plays a pivotal role in facilitating the mitosis and survival of hepatocytes; while maintaining the homeostasis of hepatocytes, and aberrant release of IL-6 can trigger inflammatory damage of the liver [32, 33]. The involvement of the IL-6 trans-signaling is indispensable for liver regeneration after partial hepatectomy. Previous studies suggested that high levels of IL-6 could potentially promote the liver regeneration pathway. However, current research indicates that low levels of IL-6 accompanied by elevated soluble IL-6 receptors can actually facilitate the IL-6 trans-signaling. It is important to note that excessive levels of IL-6 may lead to abnormal hepatocyte proliferation and exacerbate tissue damage through the classic signaling [34]. Therefore, it is still necessary to control the excessive increase of IL-6 level after surgery. In our study, it is of certain significance to select the concentration of IL-6 24 h after operation as the visual evaluation index of the prognosis of SGB after this operation. If this condition is established, it will create more favorable factors for us to apply the stellate ganglion block guided by ultrasound to a laparoscopic partial hepatectomy. After all, this is a clinically easy indicator to detect.

Regarding SGB, there is no observed impact on postoperative complications; however, a disparity in hospital stay duration exists. This discrepancy may be attributed to individual cases with prolonged hospitalization due to specific complications, although our analysis solely accounted for the number of complications. Additionally, it is plausible that the sample size of postoperative complications as a secondary outcome might have limitations. In terms of liver function comparison, no significant findings were obtained possibly due to ethical considerations influenced by the administration of liver protective drugs after surgery.

## Limitations

First, our selected patients with laparoscopic partial hepatectomy belong to the middle-aged group, and their age span is relatively large, which may have some influence on the results of postoperative inflammatory cytokines and complications. Secondly, we did not further refine the choice of laparoscopic partial hepatectomy. We included all the patients who had undergone partial hepatectomy in our hospital and met the inclusion criteria. Although this is consistent with our initial desire to study the use of ultrasound-guided stellate ganglion block in patients undergoing major surgical procedures such as laparoscopic partial hepatectomy, I believe that if we can refine the study of a small class of partial hepatectomy, we should be able to get more positive results. The third potential confounding factor in our research is that Horner syndrome will manifest itself after SGB, making it possible for patients and anesthesiologists who follow up on anesthetic work to be aware of the grouping condition.

## Conclusion

Ultrasound-guided stellate ganglion block plays a positive role in the enhanced recovery of patients after laparoscopic partial hepatectomy, which mainly shows that it can make the level of inflammatory cytokines more stable, the perioperative hemodynamics more stable, and reduce the length of stay and hospitalization expenses. The serum IL-6 level within 24 h after surgery may be used as a predictor of hospitalization.

## Data Availability

Data and materials related to this study can be obtained from the corresponding authors.
